# mGOASVM: Multi-label protein subcellular localization based on gene ontology and support vector machines

**DOI:** 10.1186/1471-2105-13-290

**Published:** 2012-11-06

**Authors:** Shibiao Wan, Man-Wai Mak, Sun-Yuan Kung

**Affiliations:** 1 Department of Electronic and Information Engineering, The Hong Kong Polytechnic University, Hong Kong SAR, China; 2 Department of Electrical Engineering, Princeton University, New Jersey, USA

## Abstract

**Background:**

Although many computational methods have been developed to predict protein subcellular localization, most of the methods are limited to the prediction of single-location proteins. Multi-location proteins are either not considered or assumed not existing. However, proteins with multiple locations are particularly interesting because they may have special biological functions, which are essential to both basic research and drug discovery.

**Results:**

This paper proposes an efficient multi-label predictor, namely mGOASVM, for predicting the subcellular localization of multi-location proteins. Given a protein, the accession numbers of its homologs are obtained via BLAST search. Then, the original accession number and the homologous accession numbers of the protein are used as keys to search against the Gene Ontology (GO) annotation database to obtain a set of GO terms. Given a set of training proteins, a set of *T* relevant GO terms is obtained by finding all of the GO terms in the GO annotation database that are relevant to the training proteins. These relevant GO terms then form the basis of a *T*-dimensional Euclidean space on which the GO vectors lie. A support vector machine (SVM) classifier with a new decision scheme is proposed to classify the multi-label GO vectors. The mGOASVM predictor has the following advantages: (1) it uses the frequency of occurrences of GO terms for feature representation; (2) it selects the relevant GO subspace which can substantially speed up the prediction without compromising performance; and (3) it adopts an efficient multi-label SVM classifier which significantly outperforms other predictors. Briefly, on two recently published virus and plant datasets, mGOASVM achieves an actual accuracy of 88.9% and 87.4%, respectively, which are significantly higher than those achieved by the state-of-the-art predictors such as iLoc-Virus (74.8%) and iLoc-Plant (68.1%).

**Conclusions:**

mGOASVM can efficiently predict the subcellular locations of multi-label proteins. The mGOASVM predictor is available online at http://bioinfo.eie.polyu.edu.hk/mGoaSvmServer/mGOASVM.html.

## Background

Knowing where a protein resides in a cell can help biologists elucidate the functions of the protein. With the accomplishment of the various large-scale genome sequencing projects, an exponentially growing number of new protein sequences have been discovered [1,2]. Computation methods are required to automatically and accurately identify the subcellular locations of these proteins.

Conventional methods for subcellular-localization prediction can be roughly divided into sequence-based methods and annotation-based methods. Sequence-based methods include: (1) sorting-signals based methods, such as PSORT [3], WoLF PSORT [4], TargetP [5] and SignalP [6,7]; (2) composition-based methods, such as amino-acid compositions (AA) [8], amino-acid pair compositions (PairAA) [8], gapped amino-acid pair compositions (GapAA) [9,10], and pseudo amino-acid composition (PseAA) [11,12]; and (3) homology-based methods, such as Proteome Analyst [13], PairProSVM [14] and other predictors [15-17].

Annotation-based methods make use of the correlation between the annotations (usually the functional annotations) of a protein and its subcellular localization. Among them, methods based on Gene Ontology (GO) information are more attractive. Gene Ontology (GO)^a^ is a set of standardized vocabularies that annotate the function of genes and gene products across different species. The term ‘ontology’ originally refers to a systematic account of existence. In the GO database, the annotations of gene products are organized in three related ontologies: cellular components, biological processes, and molecular functions. Cellular components refer to the substances that constitute cells and living organisms. Example substances are proteins, nucleic acids, membranes, and organelles. Majority of these substances are located within the cells, but there are also substances locating outside the cells (extracellular areas). A biological process is a sequence of events achieved by one or more ordered assemblies of molecular functions. A molecular function is achieved by activities that can be performed by individual or by assembled complexes of gene products at the molecular level.

As a result of the GO Consortium annotation effort, the Gene Ontology Annotation (GOA) database^b^ has become a large and comprehensive resource for proteomics research [18]. The database provides structured annotations to non-redundant proteins from many species in UniProt Knowledgebase (UniProtKB) [19] using standardized GO vocabularies through a combination of electronic and manual techniques. The large-scale assignment of GO terms to UniProtKB entries (or accession numbers) was done by converting a proportion of the existing knowledge held within the UniProKB database into GO terms [18]. The GO annotation database also includes a series of cross-references to other databases. Thus, the systematic integration of GO annotations and UniProtKB database can be exploited for subcellular localization. Specifically, given the accession number of a protein, a set of GO terms can be retrieved from the GO annotation database file^c^. In UniProKB, each protein has an accession number, and in the GO annotation database, each accession number may be associated with zero, one or more distinct GO terms. Conversely, one GO term may be associated with zero, one, or many different accession numbers. This means that the mappings between accession numbers and GO terms are many-to-many.

From the perspective of GO terms extraction, the GO-based predictors can be classified into three categories: (1) using InterProScan [20] to search against a set of protein signature databases [21-25]; (2) using the accession numbers of proteins to search against the GO annotation database such as Euk-OET-PLoc [26], Hum-PLoc [27], Euk-mPLoc [28], Gneg-PLoc [29] and an integrated method [30]; and (3) using the accession numbers of homologous proteins retrieved from BLAST [31] to search against the GO annotation database, such as ProLoc-GO [32], iLoc-Virus [33], iLoc-Gneg [34] and Cell-PLoc 2.0 [35].

However, there exist multi-location proteins that can simultaneously reside at, or move between, two or more different subcellular locations. Unfortunately, most of the existing methods are limited to the prediction of single-location proteins. These methods generally exclude the multi-label proteins or are based on the assumption that multi-location proteins do not exist. Actually, proteins with multiple locations play important roles in some metabolic processes that take place in more than one compartment, such as fatty acid *β*-oxidation in the peroxisome and mitochondria, and antioxidant defense in the cytosol, mitochondria and peroxisome [36].

There are a few predictors [33,37,38] specifically designed for predicting viral proteins, generated by viruses in various cellular compartments of the host cell or virus-infected cells. Studying the subcellular localization of viral proteins enables us to obtain the information about their destructive tendencies and consequences [33,37,38]. It is also beneficial to the annotation of the functions of viral proteins and the design of antiviral drugs. To the best of our knowledge, there are two predictors, namely Virus-mPLoc [37] and iLoc-Virus [33], capable of predicting multi-label viral proteins. iLoc-Virus performs better than Virus-mPLoc because the former has a better formulation for reflecting GO information and has a better way to predict the number of subcellular location sites of a query protein [33]. Recently, a method called KNN-SVM ensemble classifier [39] is proposed to deal with multi-label proteins, including viral proteins. It was found that the performance of the KNN-SVM predictor is comparable to iLoc-Virus and is better than Virus-mPLoc.

Conventional methods specializing for plant proteins, such as TargetP [5] and Predotar [40], can only deal with single-label proteins. Plant-mPLoc [41] and iLoc-Plant [42] are state-of-the-art predictors that can deal with single-label and multi-label proteins of plants. iLoc-Plant performs better than Plant-mPLoc because of the similar improvement as in iLoc-Virus versus Virus-mPLoc.

This paper proposes an efficient multi-label predictor, namely mGOASVM, for multi-label protein subcellular localization prediction. Here, the prefix “m” stands for multiple, meaning that the predictor can deal with proteins with multiple subcellular locations. mGOASVM is different from other predictors in that (1) it adopts a new decision scheme for an SVM classifier so that it can effectively deal with datasets containing both single-label and multi-label proteins; (2) it selects a set of distinct, relevant GO terms to form a more informative GO subspace; (3) it constructs the GO vectors by using the frequency of occurrences of GO terms instead of using 1-0 values [23,37,41] for indicating the presence or absence of some predefined GO terms. The results on two benchmark datasets and a newly created dataset full of novel proteins demonstrate that these three properties enable mGOASVM to predict multi-location proteins and outperform the state-of-the-art predictors such as iLoc-Virus and iLoc-Plant.

## Localization via direct table lookup

Because the cellular component GO terms have already been annotated with cellular component categories, it seems that what we need is to create a lookup table using the cellular component GO terms as the keys and the component categories as the hashed values. Such a naive solution, however, is undesirable, as will be explained below.

Although the cellular component ontology is directly related to the subcellular localization, we cannot simply use its GO terms to determine the subcellular locations of proteins. The reason is that some proteins do not have cellular component GO terms. Even for proteins annotated with cellular-component GO terms, it is inappropriate to use these terms only to determine their subcellular localizations. The reason is that a protein could have multiple cellular-component GO terms that map to different subcellular localizations, which are highly likely to be inconsistent with the true subcellular locations of proteins. Another reason is that proteins with annotated subcellular localization in Swiss-Prot may still be marked as ‘Cellular Component Unknown’ in the GO database [26]. Because of this limitation, it is necessary to use the other two ontologies as well because they are also relevant (although not directly) to the subcellular localization of proteins.

To exemplify the above discussion, we created a lookup table (Table 1) and developed a table-lookup procedure to predict the subcellular localization of the proteins in the virus dataset (see Table 2(a)). Table 1 has two types of GO terms: essential GO terms and child GO terms. As the name implies, the essential GO terms [32] are GO terms that are essential or critical for the subcellular localization prediction. In addition to the essential GO terms, their direct descendants (known as child terms) also possess direct localization information. The relationships between child terms and their parent terms include ‘is a’, ‘part of’ and ‘occurs in’ [43]. The former two correspond to cellular component GO terms and the third one typically corresponds to biological process GO terms. As we are more interested in cellular component GO terms, the ‘occurs in’ relationship will not be considered. For ease of reference, we refer to both essential GO terms and their child terms as ‘explicit GO terms’.

**Table 1:** Explicit GO terms for the virus dataset

**Cellular component**	**Explicit GO terms**		**No. of terms**
	**Essential GO terms**	**Child terms (Relationship)**	
		GO:00046727 (Part of), GO:0046798 (Part of),	
Viral capsid	GO:0019028	GO:0046806 (Part of), GO:0019013 (Part of),	7
		GO:0019029 (Is a), GO:0019030 (Is a)	
		GO:0044155 (Part of), GO:0044084 (Part of),	
		GO:0044385 (Part of), GO:0044160 (Is a),	
		GO:0044162 (Is a), GO:0085037 (Is a),	
		GO:0085042 (Is a), GO:0085039 (Is a),	
Host cell membrane	GO:0033644	GO:0020002 (Is a), GO:0044167 (Is a),	20
		GO:0044173 (Is a), GO:0044175 (Is a),	
		GO:0044178 (Is a), GO:0044384 (Is a),	
		GO:0033645 (Is a), GO:0044231 (Is a),	
		GO:0044188 (Is a), GO:0044191 (Is a),	
		GO:0044200 (Is a)	
Host ER^∗^	GO:0044165	GO:0044166 (Part of), GO:0044167 (Part of),	5
		GO:0044168 (Is a), GO:0044170 (Is a)	
Host cytoplasm	GO:0030430	GO:0033655 (Part of)	2
Host nucleus	GO:0042025	GO:0044094 (Part of)	2
Secreted	GO:0005576	GO:0048046 (Is a), GO:0044421 (Part of)	3

Explicit GO terms include essential GO terms and their child terms. The definition of essential GO terms can be found in [32]. Here the relationship includes ‘is a’ and ‘part of’ only, because only cellular component GO terms are analyzed here. *Relationship*: the relationship between child terms and their parent essential GO terms;

*No. of Terms*: the total number of explicit GO terms in a particular class.

^*^:host endoplasmic reticulum.

**Table 2:** Breakdown of the (a) virus protein dataset and (b) plant protein dataset

**(a) Viral protein dataset**		
**Label**	**Subcellular location**	**No. of locative proteins**
1	Viral capsid	8
2	Host cell membrane	33
3	Host endoplasmic reticulum	20
4	Host cytoplasm	87
5	Host nucleus	84
6	Secreted	20
Total number of locative proteins ( $\left(N_{\text{loc}}^{v}\right)$)		252
Total number of actual proteins ( $\left(N_{\text{act}}^{v}\right)$)		207
**(b) Plant protein dataset**		
**Label**	**Subcellular location**	**No. of locative proteins**
1	Cell membrane	56
2	Cell wall	32
3	Chloroplast	286
4	Cytoplasm	182
5	Endoplasmic reticulum	42
6	Extracellular	22
7	Golgi apparatus	21
8	Mitochondrion	150
9	Nucleus	152
10	Peroxisome	21
11	Plastid	39
12	Vacuole	52
Total number of locative proteins ( $\left(N_{\text{loc}}^{p}\right)$)		1055
Total number of actual proteins ( $\left(N_{\text{act}}^{p}\right)$)		978

For each class in Table 1, the child terms were obtained by presenting the corresponding essential GO term to the QuickGO server [44]^d^.

Given a query sequence, we first obtain its ‘GO-term’ set from the GO annotation database. Then, if one (or more than one) of the terms in this set matches an essential GO term in Table 1, the subcellular location set of this query protein is predicted to be the one (or the ones) corresponding to the matched GO term(s). For example, if the set of GO terms contains GO:0019028, then this query protein is predicted as ‘Viral capsid’; or if the set of GO terms contains both GO:0030430 and GO:0042025, then this query protein is predicted as ‘host cytoplasm’ and ‘host nucleus’. Further, if none of the terms in this set matches any essential GO terms but one (or more than one) of the terms in this set match(es) any child terms in Table 1, then the query protein is predicted as belonging to the class(es) associated with the child GO term(s). For example, if no essential GO terms can be found in the set but GO:0019030 is found, then the query protein is predicted as ‘Viral capsid’; or if GO:0044155, GO:0044166 and GO:0033655 are found, then the query protein is predicted as ‘host cell membrane’, ‘host endoplasmic reticulum’ and ‘host cytoplasm’.

A major problem of this table lookup procedure is that the GO terms of a query protein may contain many essential GO terms and/or have child terms spanning across more classes than the number of true subcellular locations, causing over-prediction. For example, in the virus dataset, 69, 14 and 3 (out of 207) proteins have explicit GO terms that map to two, three and four locations, and 121 (out of 207) proteins have explicit GO terms that map to one location. By comparing with the true locations, there are totally 139 proteins whose explicit GO terms are consistent with their true locations, of which there are 107 single-label proteins, 30 two-label proteins and 2 three-label proteins. This means that only about 67% (139/207) proteins are likely to be predicted correctly^d^. This analysis suggests that direct table lookup is not a desirable approach and this motivates us to develop machine learning methods for GO-based subcellular localization prediction.

## Results

### Datasets

In this paper, the virus dataset used in Virus-mPLoc [37] and iLoc-Virus [33] and the plant dataset used in Plant-mPLoc [41] and iLoc-Plant [42] were used to evaluate the performance of mGOASVM.

The virus dataset was created from Swiss-Prot 57.9. It contains 207 viral proteins distributed in 6 locations (see Table 2(a)). Of the 207 viral proteins, 165 belong to one subcellular locations, 39 to two locations, 3 to three locations and none to four or more locations. This means that about 20% of proteins are located in more than one subcellular location. The sequence identity of this dataset was cut off at 25%.

The plant dataset was created from Swiss-Prot 55.3. It contains 978 plant proteins distributed in 12 locations (see Table 2(b)). Of the 978 plant proteins, 904 belong to one subcellular locations, 71 to two locations, 3 to three locations and none to four or more locations. In other words, 8% of the plant proteins in this dataset are located in multiple locations. The sequence identity of this dataset was cut off at 25%.

### Performance metrics

To facilitate performance comparison, the concepts of locative proteins [33,37] and actual proteins were introduced here. If a protein exists in two different subcellular locations, it will be counted as two locative proteins; if a protein coexists in three locations, then it will be counted as three locative proteins; and so forth. But no matter how many subcellular locations a protein simultaneously resides, it will be counted as only one actual protein. Mathematically, denote *N*_loc_ as the total number of locative proteins, *N*_act_as the total number of actual proteins, *M* as the number of subcellular locations, *n*_act_(*m*) (*m*=1,…,*M*) as the number of actual proteins coexisting in *m* subcellular locations. Then, the *N*_act_ and *N*_loc_ can be expressed as:

(1) $$N_{\text{act}}=\overset{M}{\underset{m=1}{\sum }}n_{\text{act}}(m)$$

(2) $$N_{\text{loc}}=\overset{M}{\underset{m=1}{\sum }}m\cdot n_{\text{act}}(m)$$

In the virus dataset, *M*=6; and in the plant dataset, *M*=12. Then, from Eq. 1 and Eq. 2, we obtain

(3) $$N_{\text{act}}^{v}=165+39+3=207$$

(4) $$N_{\text{loc}}^{v}=1\times 165+2\times 39+3\times 3+\overset{6}{\underset{m=4}{\sum }}m\times 0=252$$

(5) $$N_{\text{act}}^{p}=904+71+3=978$$

(6) $$N_{\text{loc}}^{p}=1\times 904+2\times 71+3\times 3+\overset{12}{\underset{m=4}{\sum }}m\times 0=1055$$

where the superscript *v* and *p* stand for the virus and plant datasets, respectively. Thus, for the virus dataset, 207 actual proteins correspond to 252 locative proteins; and for the plant dataset, 978 actual proteins correspond to 1055 locative proteins. The breakdown of these two benchmark datasets were shown in Table 2(a) and Table 2(b).

In statistical prediction, leave-one-out cross validation (LOOCV) is considered to be the most rigorious and bias-free method [45]. Hence, LOOCV was used to examine the performance of mGOASVM. In each fold of LOOCV, a protein of the dataset (suppose there are *N* proteins) was singled out as the test protein and the remaining (*N*−1) proteins were used as the training data. This procedure was repeated *N* times and in each fold a different protein was selected as the test protein. This ensures that every protein in the dataset will be tested. Here, ‘proteins’ refers to ‘actual proteins’ rather than ‘locative proteins’; otherwise the training set will contain identical proteins distributed across multiple classes, which violates the SVM learning requirement that positive-class training patterns must be different from the negative-class training patterns.

The locative accuracy [46] and actual accuracy were used to measure the performance of multi-label predictors. Specifically, denote $\left(ℒ({\text{p}}_{i})\right)$ and $\left(ℳ({\text{p}}_{i})\right)$ as the true label set and the predicted label set for the *i*-th protein **p**_*i*_(*i*=1,…,*N*_act_), respectively. Then, the overall locative accuracy is:

(7) $$\Lambda_{\text{loc}}=\frac{1}{N_{\text{loc}}}\overset{N_{\text{act}}}{\underset{i=1}{\sum }}|ℳ({\text{p}}_{i})\cap ℒ({\text{p}}_{i})|$$

where |·| means counting the number of elements in the set therein and ∩ represents the intersection of sets. And the overall actual accuracy is:

(8) $$\Lambda_{\text{act}}=\frac{1}{N_{\text{act}}}\overset{N_{\text{act}}}{\underset{i=1}{\sum }}\Delta [ℳ({\text{p}}_{i}),ℒ({\text{p}}_{i})]$$

where

(9) $$\Delta [ℳ({\text{p}}_{i}),ℒ({\text{p}}_{i})]=\left\{\begin{array}{cc} 1 & \;\text{, if}\;ℳ({\text{p}}_{i})\equiv ℒ({\text{p}}_{i})\\ 0 & \;\text{, otherwise.} \end{array}\right)$$

According to Eq. 7, a locative protein is considered to be correctly predicted if any of the predicted labels matches any labels in the true label set. On the other hand, Eq. 8 suggests that an actual protein is considered to be correctly predicted only if *all* of the predicted labels match those in the true label set exactly. For example, for a protein coexist in, say, three subcellular locations, if only two of the three are correctly predicted, or the predicted result contains a location not belonging to the three, the prediction is considered to be incorrect. In other words, when and only when all the subcellular locations of a query protein are exactly predicted without any overprediction or underprediction, can the prediction be considered as correct. Therefore, the actual accuracy is stricter than the locative accuracy.

Despite its strict criteria, the actual accuracy is regarded to be more objective than the locative accuracy. Locative accuracy is liable to give biased performance measure when the predictor tends to over-predict, i.e., giving large $\left(\left|ℳ({\text{p}}_{i})\right|\right)$ for many **p**_*i*_. In the extreme case, if we predict every protein in the virus dataset to have all of the 6 subcellular locations, according to Eq. 7, the locative accuracy is 100%. But obviously, the predictions are wrong and meaningless. On the contrary, if we use the actual accuracy as the performance measure in this extreme case, the actual accuracy will be 0%, which definitely conforms to what we expect.

### Comparison with state-of-the-art predictors

Table 3(a) compares the performance of mGOASVM against two state-of-the-art virus-specialized multi-label predictors on the virus dataset. Both Virus-mPLoc [37] and iLoc-Virus [33] use the accession numbers of homologs returned from BLAST [31] as searching keys to retrieve GO terms from the GOA database. The KNN-SVM ensemble classifier [39] uses the true accession number of proteins directly as input. For a fair comparison with these two predictors, the performance of mGOASVM shown in Table 3(a) was obtained by using the accession numbers of homologous proteins as the searching keys. Like Virus-mPLoc and iLoc-Virus, mGOASVM uses BLAST [31] to find the homologs and then uses the accession numbers of the homologs as the searching keys. Here, mGOASVM selects the top homolog for each protein. If BLAST cannot find a homolog for a protein, we assign zeros to all entries of the corresponding GO vectors. In the virus dataset, a homolog can always be found for every protein.

**Table 3:** Comparing mGOASVM with state-of-the-art multi-label predictors based on leave-one-out cross validation (LOOCV) using (a) the virus dataset and (b) the plant dataset

**(a) Performance on the viral protein dataset**					
**Label**	**Subcellular location**	**LOOCV locative accuracy**			
		**Virus-mPLoc [**[37]**]**	**KNN-SVM [**[39]**]**	**iLoc-Virus [**[33]**]**	**mGOASVM**
1	Viral capsid	8/8 = 100.0%	8/8 = 100.0%	8/8 = 100.0%	8/8 = 100.0%
2	Host cell membrane	19/33 = 57.6%	27/33 = 81.8%	25/33 = 75.8%	32/33 = 97.0%
3	Host ER	13/20 = 65.0%	15/20 = 75.0%	15/20 = 75.0%	17/20 = 85.0%
4	Host cytoplasm	52/87 = 59.8%	86/87 = 98.8%	64/87 = 73.6%	85/87 = 97.7%
5	Host nucleus	51/84 = 60.7%	54/84 = 65.1%	70/84 = 83.3%	82/84 = 97.6%
6	Secreted	9/20 = 45.0%	13/20 = 65.0%	15/20 = 75.0%	20/20 = 100.0%
Overall Locative Accuracy		152/252 = 60.3%	203/252 = 80.7%	197/252 = 78.2%	244/252 = **96.8%**
Overall Actual Accuracy		–	–	155/207 =74.8%	184/207 = **88.9%**
**(b) Performance on the plant protein dataset**					
**Label**	**Subcellular location**	**LOOCV locative accuracy**			
		**Plant-mPLoc [**[41]**]**	**iLoc-Plant [**[42]**]**	**mGOASVM**	
1	Cell membrane	24/56 = 42.9%	39/56 = 69.6%	53/56 = 94.6%	
2	Cell wall	8/32 = 25.0%	19/32 = 59.4%	27/32 = 84.4%	
3	Chloroplast	248/286 = 86.7%	252/286 = 88.1%	272/286 = 95.1%	
4	Cytoplasm	72/182 = 39.6%	114/182 = 62.6%	174/182 = 95.6%	
5	Endoplasmic reticulum	17/42 = 40.5%	21/42 = 50.0%	38/42 = 90.5%	
6	Extracellular	3/22 = 13.6%	2/22 = 9.1%	22/22 = 100.0%	
7	Golgi apparatus	6/21 = 28.6%	16/21 = 76.2%	19/21 = 90.5%	
8	Mitochondrion	114/150 = 76.0%	112/150 = 74.7%	150/150 = 100.0%	
9	Nucleus	136/152 = 89.5%	140/152 = 92.1%	151/152 = 99.3%	
10	Peroxisome	14/21 = 66.7%	6/21 = 28.6%	21/21 = 100.0%	
11	Plastid	4/39 = 10.3%	7/39 = 17.9%	39/39 = 100.0%	
12	Vacuole	26/52 = 50.0%	28/52 = 53.8%	49/52 = 94.2%	
Overall Locative Accuracy		672/1055 = 63.7%	756/1055 = 71.7%	1015/1055 =**96.2%**	
Overall Actual Accuracy		–	666/978 = 68.1%	855/978 = **87.4%**	

“–” means the corresponding references do not provide the overall actual accuracy. *KNN-SVM*: the KNN-SVM ensemble classifier proposed in [39]. *Host ER*: Host endoplasmic reticulum.

Table 3(b) compares the performance of mGOASVM against two state-of-the-art plant-specialized multi-label predictors on the plant dataset. Plant-mPLoc [41] uses similar methods as Virus-mPLoc, and iLoc-Plant [42] uses similar methods as iLoc-Virus. Here mGOASVM also selects the top homolog for each protein.

As shown in Table 3, for the virus dataset, mGOASVM performs significantly better than Virus-mPLoc and iLoc-Virus; for the plant dataset, mGOASVM also performs remarkably better than Plant-mPLoc and iLoc-Plant. In the virus dataset, both the overall locative accuracy and overall actual accuracy of mGOASVM are more than 14% (absolute) higher than iLoc-Virus (96.8% vs 78.2% and 88.9% vs 74.8%, respectively); and in the plant dataset, the corresponding two measures are more than 19% (absolute) higher than iLoc-Plant (96.2% vs 71.7% and 87.4% vs 68.1%, respectively). mGOASVM also performs significantly better than KNN-SVM ensemble classifier in terms of overall locative accuracy (96.8% vs 80.7%); except for the host cytoplasm, mGOASVM is more accurate than KNN-SVM in predicting all subcellular locations. The results on both datasets demonstrate that mGOASVM is more capable of handling multi-label problems than Virus-mPLoc, iLoc-Virus, KNN-SVM ensemble classifier, Plant-mPLoc and iLoc-Plant. As for the individual locative accuracy, in the virus dataset, except for the “viral capsid” for which all of mGOASVM, Virus-mPLoc and iLoc-Virus reach 100%, the locative accuracies of mGOASVM are remarkably higher than those of Virus-mPLoc and iLoc-Virus; while in the plant dataset, the individual locative accuracies of mGOASVM for all of the 12 locations are impressively higher than those of Plant-mPLoc and iLoc-Plant.

### Kernel selection and optimization

A support vector machine (SVM) can use linear, RBF or polynomial function as its kernel. Some works [9,47] have demonstrated that RBF kernels achieve better results than linear and polynomial kernels. However, our results show that linear SVMs perform better in our case. Table 4 shows the performance of mGOASVM using different types of kernel functions with different parameters based on leave-one-out cross validation (LOOCV) using the virus dataset. For RBF SVM, the kernel parameter *σ* was selected from the set {2^−2^,2^−1^,…,2^5^}. For polynomial SVM, the degree of polynomial was set to either 2 or 3. The penalty parameter (*C*) was set to 0.1 for all cases. Table 4 shows that SVMs that use the linear kernel perform better than that with RBF and polynomial kernels. This is plausible because the dimension of GO vectors is larger than the number of training vectors, aggravating the curse of dimensionality problem in non-linear SVMs [48]. The over-fitting problem becomes more severe when the degree of non-linearity is high (small *σ*), leading to degradation in performance, as demonstrated in Table 4. In other words, highly nonlinear SVMs become vulnerable to overfitting due to the high-dimensionality of the GO vectors.

**Table 4:** Performance of mGOASVM using different kernels with different parameters based on leave-one-out cross validation (LOOCV) using the virus dataset

**Kernel**	**Parameter**	**Locative accuracy**	**Actual accuracy**
Linear SVM	–	244/252 = **96.8%**	184/207 = **88.9%**
RBF SVM	*σ*=2^−2^	182/252 = 72.2%	53/207 = 25.6%
RBF SVM	*σ*=2^−1^	118/252 = 46.8%	87/207 = 42.0%
RBF SVM	*σ*=1	148/252 = 58.7%	116/207 = 56.0%
RBF SVM	*σ*=2^1^	189/252 = 75.0%	142/207 = 68.6%
RBF SVM	*σ*=2^2^	223/252 = 88.5%	154/207 = **74.4%**
RBF SVM	*σ*=2^3^	231/252 = 91.7%	150/207 = 72.5%
RBF SVM	*σ*=2^4^	233/252 = **92.5%**	115/207 = 55.6%
RBF SVM	*σ*=2^5^	136/252 = 54.0%	5/207 = 2.4%
Polynomial SVM	*d*=2	231/252 = **91.7%**	180/207 = **87.0%**
Polynomial SVM	*d*=3	230/252 = 91.3%	178/207 = 86.0%

The penalty parameter (*C*) was set to 0.1 for all cases. *σ*is the kernel parameter for the RBF SVM; *d* is the polynomial degree in the Polynomial SVM.

### Analysis of mGOASVM

Table 5 shows the performance of the GO-vector construction methods. Linear SVMs were used in both cases, and the penalty factor was set to 0.1. The results show that term-frequency (TF) achieves a bit better performance than 1-0 value in the locative accuracy, but performs almost 2% and 7% better than 1-0 value in the actual accuracy for the virus dataset and the plant dataset, respectively, which demonstrates that the frequencies of occurrences of GO terms could also provide information for subcellular locations. The results are biologically relevant because proteins of the same subcellular localization are expected to have a similar number of occurrences of the same GO term. In this regard, the 1-0 value approach is inferior because it quantizes the number of occurrences of a GO term to 0 or 1. Moreover, the more remarkable improvement achieved for the plant dataset than that for the virus dataset also suggests that the term-frequency (TF) construction method can boost the performance more impressively for datasets with larger size and more multi-label proteins.

**Table 5:** Performance of different GO-vector construction methods based on leave-one-out cross validation (LOOCV) for (a) the virus dataset and (b) the plant dataset

**(a) Performance on the viral protein dataset**		
**GO Vector construction**	**Locative accuracy**	**Actual accuracy**
**methods**		
1-0 value	244/252 = **96.8%**	179/207 = 86.5%
Term-frequency (TF)	244/252 = **96.8%**	184/207 = **88.9%**
**(b) Performance on the plant protein dataset**		
**GO Vector construction**	**Locative accuracy**	**Actual accuracy**
**methods**		
1-0 value	1014/1055 = 96.1%	788/978 = 80.6%
Term-frequency (TF)	1015/1055 = **96.2%**	855/978 = **87.4%**

To reveal that the high locative accuracies of mGOASVM are due to the capability of mGOASVM rather than due to over-prediction, we have investigated the distributions of the number of predicted labels in both virus and plant datasets. We consider $\left(\left|ℳ({\text{p}}_{i})\right|\right)$ and $\left(\left|ℒ({\text{p}}_{i})\right|\right)$ (*i*=1,…,*N*_act_) in Eq. 7 as the number of predicted labels and the number of true labels for the *i*-th protein, respectively. The distributions of the number of labels predicted by mGOASVM are shown in Table 6. Denote $\left(n_{k}^{o}\right)$, $\left(n_{k}^{e}\right)$ or $\left(n_{k}^{u}\right)$ as the number of proteins that are over-, equal-, and under-predicted by *k* (*k*=0,…,5 for the virus dataset and *k*=0,…,11 for the plant dataset) labels. Also denote *N*^*o*^, *N*^*e*^ or *N*^*u*^ as the total number of proteins that are over-, equal-, and under-predicted, respectively. Here, over-prediction, equal-prediction and under-prediction are respectively defined as the number of predicted labels that is larger than, equal to, and smaller than the number of true labels. Table 6 shows that proteins that are over- or under-predicted account for a small percentage of the datasets only (8.7% and 1.0% over- and under-predicted in the virus dataset, 8.7% and 1.4% over- and under-predicted in the plant dataset). Even among the proteins that are over-predicted, most of them are over-predicted by one location only. These include all of the 18 proteins in the virus dataset, and 83 out of 85 in the plant dataset. None of the proteins in the virus dataset are over-predicted by more than 1 location. Only 2 out of 85 proteins in the plant dataset are over-predicted by 2 locations, and none are over-predicted by more than 2 locations. As for under-prediction, all of the under-predicted proteins are only under-predicted by 1 location in both datasets. These results demonstrate that the over-prediction and under-prediction percentages are small, which suggests that mGOASVM can effectively determine the number of subcellular locations of a query protein.

**Table 6:** Distribution of the number of labels predicted by mGOASVM for proteins in the virus and plant datasets

**Dataset**	**Condition**	**Case**	$\left(n_{k}^{o}\right)$**,**$\left(n_{k}^{e}\right)$**or**$\left(n_{k}^{u}\right)$				**(*N***^***o***^, ***N***^***e***^ or ***N***^***u***^)/***N***_**act**_
			***k*=0**	***k*=1**	***k*=2**	***k*>2**	
Virus	$\left(\left|ℳ({\text{p}}_{i})|>|ℒ({\text{p}}_{i})\right|\right)$	Over-prediction	0	18	0	0	18/207 = 8.7%
	$\left(\left|ℳ({\text{p}}_{i})|=|ℒ({\text{p}}_{i})\right|\right)$	Equal-prediction	187	0	0	0	187/207 = 90.3%
	$\left(\left|ℳ({\text{p}}_{i})|<|ℒ({\text{p}}_{i})\right|\right)$	Under-prediction	0	2	0	0	2/207 = 1.0%
Plant	$\left(\left|ℳ({\text{p}}_{i})|>|ℒ({\text{p}}_{i})\right|\right)$	Over-prediction	0	83	2	0	85/978 = 8.7%
	$\left(\left|ℳ({\text{p}}_{i})|=|ℒ({\text{p}}_{i})\right|\right)$	Equal-prediction	879	0	0	0	879/978 = 89.9%
	$\left(\left|ℳ({\text{p}}_{i})|<|ℒ({\text{p}}_{i})\right|\right)$	Under-prediction	0	14	0	0	14/978 = 1.4%

$\left(\left|ℳ(p_{i})\right|\right)$: Number of predicted labels for the *i*-th (*i*=1,…, *N*_act_) protein; $\left(\left|ℒ(p_{i})\right|\right)$: Number of the true labels for the *i*-th protein; *Over-prediction*: the number of predicted labels is larger than that of the true labels; *Equal-prediction*: the number of predicted labels is equal to that of the true labels; *Under-prediction*: the number of predicted labels is smaller than that of the true labels; $\left(n_{k}^{o},n_{k}^{e}\right)$ or $\left(n_{k}^{u}\right)$: the number of proteins that are over-, equal-, or under-predicted by *k* (*k*=0,…,5 for the virus dataset and *k=*0,…,11 for the plant dataset) labels, respectively; *N*^o^, *N*^e^ or *N*^u^: the total number of proteins that are over-, equal-, or under-predicted, respectively.

Table 7 shows the performance of mGOASVM with different inputs and different numbers of homologous proteins for the virus and plant datasets. The input data can be of three possible types: (1) accession number only, (2) sequence only and (3) both accession number and sequence. mGOASVM can extract information from these inputs by producing multiple GO vectors for each protein. Denote *#homo* as the number of homologous proteins, where *#homo*∈{0,1,2,4,8} for the virus dataset and *#homo*∈{0,1,2} for the plant dataset. For different combinations of inputs and numbers of homologs, the number of distinct GO terms can be different. Typically, the number of distinct GO terms increases with the number of homologs.

**Table 7:** Performance of mGOASVM with different inputs and different numbers of homologous proteins for (a) the virus dataset and (b) the plant dataset

**(a) Performance on the viral protein dataset**				
**Input data type**	***#homo***	***N***_***d***_***(GO)***	**Locative accuracy**	**Actual accuracy**
AC	0	331	244/252 = **96.8%**	191/207 = **92.3%**
S	1	310	244/252 = **96.8%**	184/207 = **88.9%**
S	2	455	235/252 = 93.3%	178/207 = 86.0%
S	4	664	221/252 = 87.7%	160/207 = 77.3%
S	8	1134	202/252 = 80.2%	130/207 = 62.8%
S + AC	1	334	242/252 = **96.0%**	188/207 = **90.8%**
S + AC	2	460	238/252 = 94.4%	179/207 = 86.5%
S + AC	4	664	230/252 = 91.3%	169/207 = 81.6%
S + AC	8	1134	216/252 = 85.7%	145/207 = 70.1%
**(b) Performance on the plant protein dataset**				
**Input data**	***#homo***	***N***_***d***_***(GO)***	**Locative accuracy**	**Actual accuracy**
AC	0	1532	1023/1055 = **97.0%**	863/978 = **88.2%**
S	1	1541	1015/1055 = **96.2%**	855/978 = **87.4%**
S	2	1906	907/1055 = 85.8%	617/978 = 63.1%
S + AC	1	1541	1010/1055 = **95.7%**	859/978 = **87.8%**
S + AC	2	1906	949/1055 = 90.0%	684/978 = 70.0%

*S*: Sequence; *AC*: Accession Number; *#homo*: Number of homologs used in the experiments; *N*_*d*_*(GO)*: Number of Distinct GO Terms. *#homo*=0 means only the true accession number is used.

Table 7 shows that the number of homologs can affect the performance of mGOASVM. The results are biologically relevant because the homologs can provide information about the subcellular locations. However, more homologs may bring redundant or even noisy information, which are detrimental to the prediction accuracy. For example, in the plant dataset, the performance of using one homolog is better than that of using two (87.4% vs 63.1%), which in turn suggests that we should limit the number of homologs to avoid bringing irrelevant information. Moreover, as can be seen from Table 7, the performance achieved by mGOASVM using sequences with the top homolog are comparable to that of mGOASVM using the accession number only.

Table 7 shows that mGOASVM using both sequences and accession numbers performs better than using sequences only, but worse than using accession numbers.

Table 8 shows the performance of mGOASVM for multi-location proteins using different inputs. Denote *l* ( $\left(l=1,\ldots ,ℒ\right)$) as the number of co-locations. As the maximum number of co-locations in both datasets is 3, the individual actual accuracies for *l* (*l*=1,…,3) are shown in Table 8. Note that high actual accuracies for *l*>1 are more difficult to achieve than that for *l*=1, since not only the number of subcellular locations for a protein should be predicted correctly, but also the subcellular locations should be predicted precisely. As can be seen, mGOASVM achieves high performance not only for single-label proteins (the column corresponding to *l*=1), but also for multi-label proteins (the columns corresponding to *l*=2 and *l*=3). The results demonstrate that mGOASVM can tackle multi-label problems well.

**Table 8:** Performance of mGOASVM on (a) the virus dataset and (b) the plant dataset

**(a) Performance on the viral protein dataset**					
**Input data**	***#homo***	**Actual accuracy of protein groups**			**Overall actual accuracy**
		***l*=1**	***l*=2**	***l*=3**	
AC	0	154/165 = 93.3%	34/39 = 87.2%	3/3 = 100%	191/207 = **92.3%**
S	1	148/165 = 89.7%	33/39 = 84.6%	3/3 = 100%	184/207 = 88.9%
S + AC	1	151/165 = 91.5%	34/39 = 87.2%	3/3 = 100%	188/207 = 90.8%
**(b) Performance on the plant protein dataset**					
**Input data**	***#homo***	**Actual accuracy of protein groups**			**Overall actual accuracy**
		***l*=1**	***l*=2**	***l*=3**	
AC	0	813/904 = 89.9%	49/71 = 69.0%	1/3 = 33.3%	863/978 = **88.2%**
S	1	802/904 = 88.7%	52/71 = 73.2%	1/3 = 33.3%	855/978 = 87.4%
S + AC	1	811/904 = 89.7%	47/71 = 66.2%	1/3 = 33.3%	859/978 = 87.8%

*S*: Sequence; *AC*: Accession Number; *#homo*: Number of homologs used in the experiments; *l* (*l*=1,…,3): Number of co-locations. *#homo*=0 means only the true accession number is used.

## Prediction of novel proteins

### Dataset construction

To further demonstrate the effectiveness of mGOASVM, a plant dataset containing novel proteins was constructed by using the criteria specified in [41,42]. The complete procedures of constructing the novel dataset can be found in the mGOASVM web-server. Specifically, to ensure that the proteins are really novel to mGOASVM, the creation dates of these proteins should be significantly later than the training proteins (from the plant dataset) and also later than the GOA database. Because the plant dataset was created in 2008 and the GOA database used was released on 08-Mar-2011, we selected the proteins that were added to Swiss-Prot between 08-Mar-2011 and 18-Apr-2012. Moreover, proteins with multiple subcellular locations that falls within the 12 classes specified in Table 2(b) were included. After limiting the sequence similarity to 25%, 175 plant proteins distributed in 12 subcellular locations (see Table 9) were selected. Of the 175 plant proteins, 147 belong to one subcellular location, 27 belong to two locations, 1 belong to three locations and none to four or more locations. In other words, 16% of the plant proteins in this novel dataset are located in multiple locations. The protein sequences of this new dataset can be donwloaded from the mGOASVM server.

**Table 9:** Breakdown of the new plant dataset

**Label**	**Subcellular location**	**No. of locative proteins**
1	Cell membrane	16
2	Cell wall	1
3	Chloroplast	54
4	Cytoplasm	38
5	Endoplasmic reticulum	9
6	Extracellular	3
7	Golgi apparatus	7
8	Mitochondrion	16
9	Nucleus	46
10	Peroxisome	6
11	Plastid	1
12	Vacuole	7
Total number of locative proteins		204
Total number of actual proteins		175

The dataset was constructed from Swiss-Prot created between 08-Mar-2011 and 18-Apr-2012. The sequence identity of the dataset is below 25%.

### Prediction procedure

**Figure 1:**
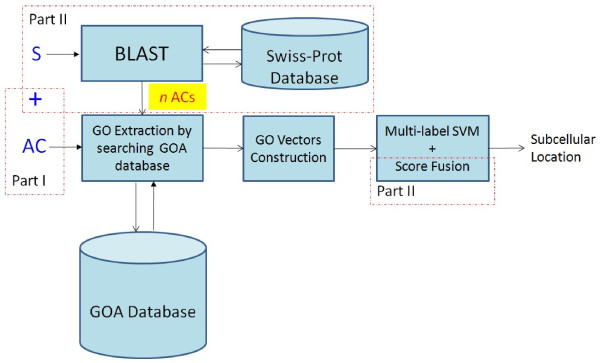
**Flowchart of mGOASVM for three cases: (1) using accession numbers only; (2) using sequences only; (3) using both accession numbers and sequences.** AC: Accession Number; S: Sequence. Part II does not exist for Case 1, and Part I does not exist for Case 2. Case 3 requires using both Part I and Part II. The score fusion implements Eq. 12.

Because the novel proteins were recently added to Swiss-Prot, many of them have not been annotated in the GOA database. As a results, if we used the accession numbers of these proteins to search against the GOA database, the corresponding GO vectors will contain all zeros. This suggests that we should use the ACs of their homologs as the searching keys, i.e., the procedure shown in Figure 1 using sequences as input should be adopted. However, we observed that for some novel proteins, even the top homologs do not have any GO terms annotated to them. To overcome this limitation, the following procedure was adopted. For the proteins whose top homologs do not have any GO terms in the GOA database, we used the second-top homolog to find the GO terms; similarly, for the proteins whose top and 2-nd homologs do not have any GO terms, the third-top homolog was used; and so on until all the query proteins can correspond to at least one GO term. In the case where BLAST fails to find any homologs, we used the method PseAA [11] as a back-up. In this dataset, among 175 proteins, 5 of them require to use the backup method.

Because BLAST searches were used in the above procedure, the prediction performance will depend on the closeness (degree of homology) between the training proteins and test proteins. To determine the number of test proteins that are close homologs of the training proteins, we performed a BLAST search for each of the test proteins. The E-value threshold was set to 10 so that none of the proteins in the lists returned from BLAST have E-value larger than 10. Then, we identified the training proteins in the lists based on their accession numbers, and recorded their corresponding E-values.

**Figure 2:**
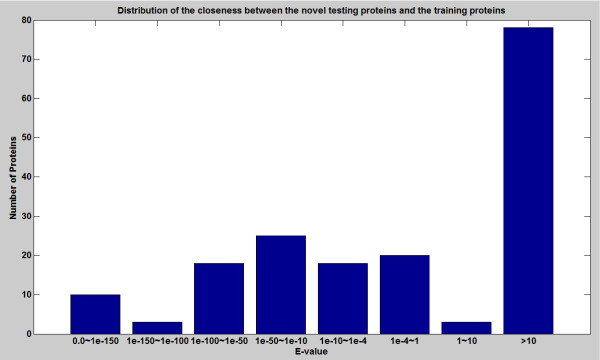
**Distribution of the closeness between the novel testing proteins and the training proteins.** The *closeness* is defined as the BLAST E-values of the training proteins using the test proteins as the query proteins in the BLAST searches. *Number of Proteins*: The number of training proteins whose E-values fall into the interval specified under the bar. Small E-values suggest that the corresponding novel proteins are close homologs of the training proteins.

Figure 2 shows the distribution of the E-values, which quantify the closeness between the training proteins and test proteins. If we use a common criteria that homologous proteins should have E-value less than 10^−4^, then 74 out of 175 test proteins are homologs of training proteins, which account for 42% of the test set. Note that this homologous relationship does not mean that using BLAST’s homology transfers can predict all of the test proteins correctly. In fact, BLAST’s homology transfers (based on the CC field of the homologous proteins) can only achieve a prediction accuracy of 26.9% (47/175). As the prediction accuracy of mGOASVM on this test set (see Table 10) is significantly higher than this percentage, the extra information available from the GOA database plays a very important role in the prediction.

**Table 10:** Comparing mGOASVM with a state-of-the-art multi-label plant predictor based on independent tests using the new plant dataset

**Label**	**Subcellular location**	**Independent test locative accuracy**	
		**Plant-mPLoc [**[41]**]**	**mGOASVM**
1	Cell membrane	8/16 = 50.0%	7/16 = 43.8%
2	Cell wall	0/1 = 0%	0/1 = 0%
3	Chloroplast	27/54 = 50.0%	39/54 = 72.2%
4	Cytoplasm	5/38 = 13.2%	19/38 = 50.0%
5	Endoplasmic reticulum	1/9 = 11.1%	3/9 = 33.3%
6	Extracellular	0/3 = 0%	1/3 = 33.3%
7	Golgi apparatus	3/7 = 42.9%	3/7 = 42.9%
8	Mitochondrion	6/16 = 37.5%	11/16 = 68.8%
9	Nucleus	31/46 = 67.4%	33/46 = 71.7%
10	Peroxisome	4/6 = 66.7%	3/6 = 50.0%
11	Plastid	0/1 = 0%	0/1 = 0%
12	Vacuole	2/7 = 28.6%	4/7 = 57.1%
Overall locative accuracy		87/204 = 42.7%	123/204 =**60.3%**
Overall actual accuracy		60/175 = 34.3%	97/175 = **55.4%**

### Prediction performance

Table 10 shows the prediction performance of mGOASVM on this novel protein dataset. As explained earlier, to ensure that these proteins are novel to mGOASVM, 978 proteins of the plant dataset (See Table 2(b)) were used for training the classifier. We compared mGOASVM with the Plant-mPLoc [41] web-server^e^. As shown in Table 10, mGOASVM performs significantly better than Plant-mPLoc. The overall locative accuracy and the overall actual accuracy of mGOASVM are more than 17%, 21% higher than those of Plant-mPLoc, respectively (locative accuracy 60.3% vs 42.7%, and actual accuracy 55.4% vs 34.3%). For most of 12 individual subcellular locations, mGOASVM outperforms Plant-mPLoc, except in cell membrane and peroxisome. Given the novelty and multi-label properties of these proteins and the low sequence similarity (below 25%), the locative accuracy of 60.3% and the actual accuracy of 55.4% achieved by mGOASVM are fairly high. On the other hand, due to the scarcity of data, mGOASVM does not perform well in some subcellular locations, such as cell wall and plastid. But the situation will be improved when more and more proteins are available for training our SVM classifiers.

## Discussion

mGOASVM possesses several desirable properties that make it outperform Virus-mPLoc [37], iLoc-Virus [33], Plant-mPLoc [41] and iLoc-Plant [42], which are specified subsequently.

### GO-Vector construction

Virus-mPLoc and Plant-mPLoc construct GO vectors by using 1-0 value to indicate the presence and absence of some predefined GO terms. This method is simple and logically plausible, but some information will be inevitably lost because it quantizes the frequency of occurrences of GO terms to either 1 or 0. The GO vectors in iLoc-Virus and iLoc-Plant contain more information than those in Virus-mPLoc and Plant-mPLoc, because the former two consider not only the GO terms of the query protein but also the GO terms of its homologs. Specifically, instead of using 1-0 value, each element of the GO vectors in the iLoc-Virus and iLoc-Plant represents the percentage of homologous proteins containing the corresponding GO term. However, the method ignores the fact that a GO term may be used to annotate the same protein multiple times under different entries in the GO annotation database. On the contrary, mGOASVM uses the frequency of occurrences of GO terms to construct the GO vectors. Intuitively, this is because proteins of the same subcellular localization tend to be annotated by similar sets of GO terms. The advantages of using the GO term-frequency count as features is evident by the superior results in Table 5.

### Capability of handling multi-label problems

An efficient way to handle multi-label problems is to predict the number of labels for each sample first, and then to predict the specific label set for each sample according to the order of the scores. Let us compare mGOASVM with two kinds of existing approaches. 

• When predicting the number of subcellular locations for a query protein, iLoc-Virus and iLoc-Plant determine the number of labels of a query protein based on the number of labels of its nearest training sample. mGOASVM, on the contrary, determines the number of labels for a query protein by looking at the number of positive-class decisions among all of the one-vs-rest SVM classifiers. Therefore, the number of labels depends on the whole training set as opposed to the query protein’s nearest neighbor in the training set.

• As opposed to Virus-mPLoc and Plant-mPLoc which require a pre-defined threshold, our mGOASVM adopts a machine learning approach to solving the multi-label classification problem. The predicted class labels in mGOASVM are assigned based on the SVMs that produce positive responses to the query protein.

In summary, the superiority of mGOASVM in handling multi-label problems is evident in Table 3.

From the machine learning perspective, prediction of multi-location proteins is a multi-label learning problem. Approaches to addressing this problem can be divided into types: problem transformation and algorithm adaptation [49]. The multi-label KNN classifiers used in iLoc-Plant and iLoc-Virus belong to the first type whereas our multi-label SVM classifier belongs to the second type. While our results show that multi-label SVMs perform better than multi-label KNN, further work needs to be done to compare these two types of approaches in the context of multi-label subcellular localization.

### GO subspace selection

To facilitate the sophisticated machine learning approach for the multi-label problem, GO subspace selection is adopted. Unlike the traditional methods [33,37,41,42] which use all of the GO terms in the GO annotation database to form the GO-vector space, mGOASVM selects a relevant GO subspace by finding a set of distinct, relevant GO terms. With the rapid growth of the GO database, the number of GO terms is also increasing. As of March 2011, the number of GO terms is 18656, which means that without feature selection, the GO vectors will have dimension 18656. This imposes computational burden on the classifier, especially when leave-one-out cross validation is used for evaluation. There is no doubt that many of the GO terms in the full space are redundant, irrelevant or even detrimental to prediction performance. By selecting a set of distinct GO terms to form a GO subspace, mGOASVM can reduce the irrelevant information and at the same time retain useful information. As can be seen from Table 7, for the virus dataset, around 300 to 400 distinct GO terms are sufficient for good performance. Therefore, using GO subspace selection can tremendously speed up the prediction without compromising the performance.

## Conclusions

This paper proposes an efficient multi-label predictor – mGOASVM – to predict the subcellular locations of multi-label proteins. By using the accession numbers of query proteins as the searching keys to search against the GO annotation database, the GO terms of each query protein are retrieved. For proteins without an accession number, BLAST is used to find their homologs and the accession numbers of the homologs are used as the searching keys. Then the GO terms are used to construct the GO vectors, which are subsequently recognized by support vectors machine (SVM) classifiers equipped with a decision strategy that can produce multiple class labels for a query protein.

Comparing with the exsiting predictors, mGOASVM has the following advantages: (1) the improved SVM classifier used in mGOASVM can effectively deal with multi-label problems; (2) it selects a relevant GO subspace from the full GO vector space by using a set of distinct GO terms; and (3) it constructs the GO vectors by using the frequency of occurrences of GO terms instead of using 1-0 values for indicating the presence or absence of some predefined GO terms.

Experimental results demonstrate that mGOASVM can efficiently predict the subcellular locations of multi-label proteins. This work also demonstrates that it is not necessary to use a large number of homologous accession numbers for searching the GO annotation database. In fact, our results strongly suggest that using the top homologous accession number is already sufficient. For readers’ convenience, mGOASVM is available online at http://bioinfo.eie.polyu.edu.hk/mGoaSvmServer/mGOASVM.html.

## Methods

The mGOASVM predictor uses accession numbers, amino acid (AA) sequences, and combination of both as input. The prediction process is divided into three parts: (1) GO terms extraction, (2) GO-vector construction and (3) multi-label one-vs-rest SVM classification.

### Retrieval of GO terms

Given a query protein, mGOASVM can handle three possible cases: (1) only the accession number is known, (2) only the amino acid sequence is known, and (3) both accession number and amino acid sequences are known. For proteins with known accession numbers, their respective GO terms are retrieved from the GOA database using the accession numbers as the searching keys. For a protein without an accession number, its amino acid sequence is presented to BLAST [31] to find *n* homologs, whose accession numbers are then used as keys to search against the GOA database. This results in *n* sets of GO terms, one set for each homologous accession number. For a protein with both accession number and amino acid sequence, the accession number and the accession numbers of its homologs are used as searching keys, resulting in (*n* + 1) sets of GO terms.

In this work, we considered different numbers of homologs (1, 2, 4, and 8 for the virus dataset, and 1 and 2 for the plant dataset) to investigate how the number of homologs affects the prediction performance.

Note that the gene association file^f^ that we downloaded from the GOA database does not provide any subcellular localization labels. This file only allows us to create a hash table storing the association between the accession numbers and their corresponding GO terms. This hash table covers all of the accession numbers in the GOA database released on 08-Mar-2011, meaning that it will also cover the 207 accession numbers in the virus dataset (dated 22-Sept-2009) but not the accession numbers in the new plant dataset. It is important to emphasize that given a query protein, having a match in this hash table does not mean that a subcellular-localization assignment can be obtained. In fact, having a match only means that a non-null GO vector can be obtained. After that, the SVMs play an important role in classifying the non-null GO vector.

### Construction of GO vectors

Given a dataset, we used the procedure described in the last subsection to retrieve the GO terms of all of its proteins. Then, we determined the number of distinct GO terms corresponding to the dataset. Suppose *T* distinct GO terms were found; these GO terms form a GO Euclidean space with *T* dimensions. For each protein in the dataset, we constructed a GO vector by matching its GO terms to all of the *T* GO terms. We have investigated two approaches to determine the elements of the GO vectors. 

1. **1-0 value**. In this approach, each of the *T* GO terms represents one canonical basis of a Euclidean space, and a protein is represented by a point in this space with coordinates equal to either 0 or 1. Specifically, the GO vector of the *i*-th protein is denoted as:

(10) $${\text{p}}_{i}=\left[\begin{array}{c} a_{i,1}\\ \vdots \\ a_{i,j}\\ \vdots \\ a_{i,T} \end{array}\right]\text{where}\;a_{i,j}=\left\{\begin{array}{cc} 1 & \;\text{, GO hit}\\ 0 & \;\text{, otherwise} \end{array}\right)$$

where ‘GO hit’ means that the *j*-th GO term appears in the GO term search result using the accession number of the *i*-th protein or its homolog(s) as the searching key.

2. **Term-Frequency (TF)**. This approach is similar to the 1-0 value approach in that a protein is represented by a point in the *T*-dim Euclidean space. However, unlike the 1-0 approach [37,41] , it uses the number of occurrences of individual GO terms as the coordinates. Specifically, the GO vector **p**_*i*_ of the *i*-th protein is defined as:

(11) $${\text{p}}_{i}=\left[\begin{array}{c} b_{i,1}\\ \vdots \\ b_{i,j}\\ \vdots \\ b_{i,T} \end{array}\right]\text{where}b_{i,j}=\left\{\begin{array}{cc} f_{i,j} & \;\text{, GO hit}\\ 0\;\; & \;\text{, otherwise} \end{array}\right)$$

where *f*_*i*,*j*_ is the number of occurrences of the *j*-th GO term (term-frequency) in the *i*-th protein. The rationale is that the term-frequencies may also contain important information for classification and therefore should not be quantized to either 0 or 1. Note that *b*_*i*,*j*_’s are analogous to the term-frequencies commonly used in document retrieval.

### Multi-label multi-class SVM classification

To predict the subcellular locations of datasets containing both single-label and multi-label proteins, a multi-label support vector machine (SVM) classifier is proposed in this paper. GO vectors are used for training the multi-label one-vs-rest SVMs. Specifically, for an *M*-class problem (here *M* is the number of subcellular locations), *M* independent binary SVMs are trained, one for each class. Denote the GO vector created by using the true accession number of the *i*-th query protein as **q**_*i*,0_and the GO vectors created by using the *n* homologous accession numbers as **q**_*i*,*j*_, *j*=1,…,*n*. Then, the score of the *m*-th SVM given the *i*-th query protein is

(12) $$s_{m}({\text{q}}_{i})=\overset{n}{\underset{j=0}{\sum }}w_{j}\left(\underset{r\in S_{m}}{\sum }\alpha_{m,r}y_{m,r}K({\text{p}}_{r},{\text{q}}_{i,j})+b_{m}\right)$$

where $\left(S_{m}\right)$ is the set of support vector indexes corresponding to the *m*-th SVM, *α*_*m*,*r*_are the Lagrange multipliers, *K*(·,·) is a kernel function, and *w*_*j*_’s are fusion weights such that $\left(\overset{n}{\underset{j=0}{\sum }}w_{j}=1\right)$. In this work, linear kernels were used, i.e., *K*(**p**_*r*_,**q**_*i*,*j*_)=〈**p**_*r*_,**q**_*i*,*j*_〉. *y*_*m*,*r*_∈{−1, + 1} are the class labels (here we call them “the transformed labels”), which are denoted as: 

1. For single-label **p**_*r*_,

(13) $$y_{m,r}=\left\{\begin{array}{cc} +1 & \;\text{, if}\;ℒ({\text{p}}_{r})=m\\ -1 & \;\text{, otherwise.} \end{array}\right)$$

2. For multi-label **p**_*r*_,

(14) $$y_{m,r}=\left\{\begin{array}{cc} +1 & \;\text{, if}\;ℒ({\text{p}}_{r})⋂\{m\}\neq \emptyset \\ -1 & \;\text{, otherwise.} \end{array}\right)$$

where *m*∈{1,…,*M*}. Note that unlike the single-label problem where each protein has one and only one positive transformed label, a multi-label protein can have more than one positive transformed label.

Then the subcellular location(s) of the *i*-th query protein will be predicted as:

(15) $${ℳ}^{*}({\text{q}}_{i})={⋃}_{m=1}^{M}\{m:s_{m}({\text{q}}_{i})>0\}.$$

As can be seen, $\left({ℳ}^{*}({\text{q}}_{i})\right)$ is a predicted set that may have zero, one, or more than one element, which enables us to make multi-label prediction. In case Eq. 15 does not produce a class label, i.e., $\left({ℳ}^{*}({\text{q}}_{i})=\emptyset \right)$, the number of subcellular locations is set to one and the location is given by

(16) $${ℳ}^{*}({\text{q}}_{i})={\text{arg max}}_{m=1}^{M}s_{m}({\text{q}}_{i}).$$

Note that **p**_*r*_’s in Eq. 12 represents the GO training vectors, which may include the GO vectors created by using the true accession numbers of the training proteins or their homologous accession numbers. We have the following three cases. 

1. If only the true accession numbers are available, then only **q**_*i,0*_’s exist and **p**_*r*_’s represent the GO training vectors created by using the true accession numbers only. In that case, **q**_*i*,*j*_(*j*=1,…,*n*) do not exist; *w*_0_=1 and *w*_*j*_=0 (*j*=1,…,*n*). Then, Eq 12 can be written as:

(17) $$s_{m}({\text{q}}_{i})=\underset{r\in S_{m}}{\sum }\alpha_{m,r}y_{m,r}K({\text{p}}_{r},{\text{q}}_{i,0})+b_{m}.$$

2. If only the amino acid sequences are known, then only the accession numbers of the homologous sequences can be used for training the SVM and for scoring. In that case, **q**_*i*,0_ does not exist and *w*_0_=0; moreover, **p**_*r*_’s represent the GO training vectors created by using the homologous accession numbers only.

3. If both accession numbers and amino acid sequences are known, then both true accession numbers and the accession numbers of the homologous sequences are used for training the SVM and for scoring. Then, **q**_*i*,*j*_(*j*=0,…,*n*) exist, and **p**_*r*_’s represent the GO training vectors created by using both the true accession numbers and the homologous accession numbers.

In this work, 1, 2, 4 and 8 homologs were tried for the virus dataset, and 1 and 2 homologs were used for the plant dataset, respectively, i.e., *n*∈{1,2,4,8} and *n*∈{1,2} in Eq. 12, respectively. For convenience, equal weights for the true accession number and the accession numbers of homologs were adopted. Therefore, for Case 2, *w*_0_=0 and *w*_*j*_=1/*n*, *j*=1,…,*n*; and for Case 3, we set *w*_*j*_=1/(*n* + 1), *j*=0,…,*n*.

Figure 1 illustrates the whole prediction process in mGOASVM for all the three cases: (1) using accession numbers only, (2) using sequences only and (3) using both accession numbers and sequences. Part II does not exist for Case 1, and Part I does not exist for Case 2. Both Part I and Part II exist for Case 3. Score fusion is the fusion of GO scores obtained from accession numbers of homologs in Case 2 or from both true accession numbers and accession numbers of homologs in Case 3.

## Endnotes

^a^http://www.geneontology.org^b^http://www.ebi.ac.uk/GOA^c^ftp://ftp.ebi.ac.uk/pub/databases/GO/goa/UNIPROT/^d^In our method, we have more than 300 relevant GO terms for the virus dataset. Even for such a small number of explicit GO terms, many proteins have explicit GO terms spanning several classes.^e^The iLoc-Plant [42] web-server is not working properly during testing; so we only reported the performance of the Plant-mPLoc [41] web-server.^f^ftp://ftp.ebi.ac.uk/pub/databases/GO/goa/UNIPROT/gene_association.goa_uniprot.gz

## Competing interests

The authors declare that they have no competing interests.

## Authors’ contributions

WSB designed the system, implemented programs and participated in manuscript preparation. MMW carried out the analysis, reviewed the study and participated in manuscript preparation. KSY supervised the whole project and participated in manuscript preparation. WSB and MMW conceived the idea of this work. All authors read and approved the final manuscript.
